# Sacral Insufficiency Fracture after Radiotherapy for Cervical Cancer: Appearance and Dynamic Changes on 18F-Fluorodeoxyglucose Positron Emission Tomography/Computed Tomography

**DOI:** 10.1155/2021/5863530

**Published:** 2021-11-22

**Authors:** Yu Ji, Chunchun Shao, Yong Cui, Dai Shi, Na Su, Yaru Wang, Jingsong Zheng

**Affiliations:** ^1^Department of Radiology, The Second Hospital, Cheeloo College of Medicine, Shandong University, 247 Beiyuan Rd, 250033 Jinan, Shandong, China; ^2^Department of Evidence-Based Medicine, The Second Hospital, Cheeloo College of Medicine, Shandong University, 247 Beiyuan Rd, 250033 Jinan, Shandong, China; ^3^Department of Nuclear Medicine, Zhongshan Hospital, Fudan University, No. 180 Fenglin Road Xuhui District, Shanghai 200032, China; ^4^Department of PET/CT, Shandong Cancer Hospital and Institute, Shandong First Medical University and Shandong Academy of Medical Sciences, 440 Jiyan Road, 250117 Jinan, Shandong, China

## Abstract

**Objective:**

With the increasing application of radiotherapy for cervical cancer, the incidence of sacral insufficiency fracture (SIF) is increasing gradually. Incorrect or untimely treatment caused by misdiagnosis may lead to serious adverse clinical consequences. This study retrospectively analyzed SIF caused by radiotherapy regarding the appearance and dynamic changes in 2-[fluorine-18]-fluoro-2-deoxy-D-glucose (18F-FDG) positive emission tomography (PET)/computed tomography (CT) images to improve the understanding of SIF.

**Materials and Methods:**

We retrospectively examined cervical cancer patients who underwent pelvic radiotherapy and 18F-FDG PET/CT between January 2014 and January 2021. Comparative analysis of the imaging performance and follow-up data was conducted. In total, 38 patients with ages ranging from 28 to 81 years (mean age 59.2 ± 10.6 y, median age 56 y) participated in the study. The respective characteristics of the 38 patients were summarized, and diagnosis was confirmed by follow-up changes.

**Results:**

Twenty-five (65.8%) of the 38 patients suffered from unilateral SIF, and 13 (34.2%) suffered from bilateral SIF. After receiving radiotherapy, SIF first appeared in 3–42 months (median, 13 months). The main 18F-FDG PET/CT manifestations of SIF were increased bone density (35/38, 92.1%), anterior sacral fracture line (28/38, 73.7%), and diffuse or linear uptake patterns parallel to the sacroiliac joint (37/38, 97.3%), with the maximum standard uptake value (SUVmax) ranging from 1.8 to 5.9 (average, 3.1). Follow-up lasted 3–59 months (mean, 14 months). The main changes in SIF were increases in the bone density and high-density range and decreases in the FDG uptake intensity and hypermetabolism range. Three patients had secondary sacral or sacroiliac joint infection (3/38, 7.9%), and 3 patients had secondary fracture and/or pelvic deformation (3/38, 7.9%).

**Conclusions:**

18F-FDG PET/CT is an effective technique for diagnosing SIF. A small fracture line in the anterior sacrum and diffuse or linear areas of high density or metabolism parallel to the sacroiliac joint were the characteristic features of SIF. The main changes in SIF were increases in the bone density and high-density range and decreases in the FDG uptake intensity and hypermetabolism range.

## 1. Introduction

Insufficiency fracture (IF) is a stress fracture caused by normal or physiological stress acting on bone affected by demineralization or reduced elasticity [1–3]. The sacrum, which is part of the posterior ring of the pelvis, is the most common site of IF; the sacrum conducts most of the gravitational force of the body and is an area of bone stress concentration [4]. There are many causes of IF, such as osteoporosis, radiotherapy, rheumatoid arthritis, and hyperparathyroidism [1, 5, 6]. Radiotherapy is the main treatment for many pelvic tumors. Although radiotherapy effectively improves the survival of patients, it is also the most common cause of sacral insufficiency fracture (SIF) [7, 8]. SIF often causes lower back pain, which is easily misdiagnosed as bone metastasis in cancer patients, and leads to unnecessary biopsy or antitumor treatment. Delayed diagnosis and treatment of SIF can also lead to other serious complications (such as infection and paralysis) [9]. Therefore, clinical and imaging physicians should deepen their understanding of SIF to avoid missed and erroneous diagnoses.

The main pathological manifestations of SIF are repeated microfractures and repair in cancellous bone, which increase glucose uptake. 2-[Fluorine-18]-fluoro-2-deoxy-D-glucose (18F-FDG) positive emission tomography (PET) can detect this change sensitively. Meanwhile, low-dose computed tomography (CT) can show the density and morphological characteristics of bone lesions. Therefore, PET/CT is an effective tool for detecting SIF. As 18F-FDG PET/CT is widely used in the staging and monitoring of malignant tumors, physicians are facing new challenges: what is the performance of 18F-FDG PET/CT in detecting SIF after radiotherapy? How can SIF be effectively distinguished from malignant bone metastasis in PET/CT images? Although previous studies have described the performance of bone scans, CT, and magnetic resonance imaging (MRI) in detecting SIF [1, 10, 11], only a few studies have discussed the 18F-FDG PET/CT manifestations of SIF [12–14], and no studies have described the dynamic features of SIF. This study was jointly conducted by two hospitals in a large number of patients. According to the needs of tumor diagnosis and treatment, patients often require multiple 18F-FDG PET/CT and CT reexaminations, which provide convenient conditions for dynamic observation of the disease.

In this study, we aimed to determine the 18F-FDG PET/CT imaging characteristics and dynamic changes in SIF. To achieve this goal and improve the understanding of SIF, we retrospectively analyzed the imaging data of all the patients with cervical cancer receiving pelvic radiotherapy and summarized the 18F-FDG PET/CT manifestations and changes in SIF.

## 2. Materials and Methods

### 2.1. Patients

This study was a retrospective analysis of the data of cervical cancer patients undergoing 18F-FDG PET/CT examination at Shandong Cancer Hospital and Institute and the Second Hospital of Shandong University from January 2014 to January 2021. Thirty-eight patients with complete follow-up data were finally included in the study, ranging in age from 28 to 81 years (mean age 59.2 ± 10.6 y, median age 56 y). The demographic and clinical data of all the patients are shown in Table 1. The protocol of this study was approved by the local ethics committee for retrospective analyses. This study was approved by the Shandong Cancer Hospital and Institute and the Second Hospital of Shandong University Review Boards for clinical investigation. All of the methods were performed in accordance with the Declaration of Helsinki and the relevant guidelines. Due to the retrospective nature of the study, informed consent was waived.

The criteria for inclusion in this study were as follows: (1) complete clinical data of patients with cervical cancer; (2) a history of pelvic radiotherapy; (3) vaseline 18F-FDG PET/CT or CT data from before radiotherapy; and (4) 18F-FDG PET/CT data obtained at least once after SIF was diagnosed by 18F-FDG PET/CT. The diagnostic criteria of SIF were as follows: (1) compared with baseline CT, new fracture lines and/or sclerosis of the sacrum and (2) increased metabolism in the sacral region and no evidence of bone metastases during follow-up (such as osteolytic bone destruction and soft tissue mass).

### 2.2. Scan Technique

Imaging of patients was conducted on a PET/CT scanner (TF Big Bore, Philips; and Ingenuity TF, Philips). 18F-FDG with a pH of 5–7 and a radiochemical purity exceeding 95% was produced using a cyclotron (MINItrace, GE Healthcare). The patients underwent fasting for at least 6 h and had blood glucose levels below 200 mg/dL prior to injection with 18F-FDG. Patients were required to lay in a quiet room for 60 min after intravenous injection with 4.4–5.5 MBq/kg 18F-FDG.

Spiral CT scanning was performed at 120 kVp and 300 mA·s. PET was performed after spiral CT without patient repositioning. PET images were obtained at 7 to 8 couch positions per patient, with an acquisition time of 1.5 min per position. We used CT scan data for attenuation correction of PET images and then fused the attenuation-corrected PET and CT images.

### 2.3. Follow-Up

All the patients included in this study underwent at least one pelvic baseline CT examination before radiotherapy and 18F-FDG PET/CT examination for the initial discovery of SIF. After SIF diagnosis, at least one follow-up 18F-FDG PET/CT was performed. The follow-up period was at least 3 months to ensure the accuracy of the diagnosis. Since the SIF follow-up time was based on the tumor follow-up time, it was difficult to manage the follow-up time uniformly.

### 2.4. Image Analysis

The images were reviewed by three readers (2 nuclear medicine physicians and 1 radiologist): reader 1 was an attending with 15 y of experience in PET/CT diagnosis; reader 2 was an attending with 10 y of experience in PET/CT diagnosis; and reader 3 was an attending with 25 y of experience in bone imaging. They were blinded to the original study interpretation and report but in knowledge of the clinical tumor. Each reader reviewed the images separately in a blinded manner, and differences were resolved by consensus. The main purpose of this study was to describe the PET/CT findings and dynamic changes of SIF; thus, to ensure the accuracy of the study, if readers could not resolve discrepancies in the imaging results, the case was excluded.

We conducted a visual and semiquantitative analysis of SIF features. The pattern of FDG uptake in SIF patients was evaluated by visual analysis and compared with baseline CT and surrounding normal bone before radiotherapy to determine whether there were new bone abnormalities. In the semiquantitative analysis, to reduce the interference of other factors with the maximum standard uptake value (SUVmax), we used the target-to-background ratio (T/B = lesion/liver) as a method to evaluate the dynamic changes in metabolism, using a 10% change in T/B on follow-up as the threshold. For patients with multiple follow-ups, the imaging data and T/B of the last follow-up were considered.

### 2.5. Statistical Analysis

All the data are expressed as the mean ± standard deviation. The clinical data of the patients and scan results were analyzed using descriptive statistics, including frequencies, means, and medians. All statistical analyses were performed in SPSS ver. 23.0 for Windows.

## 3. Results

**Figure 1:**
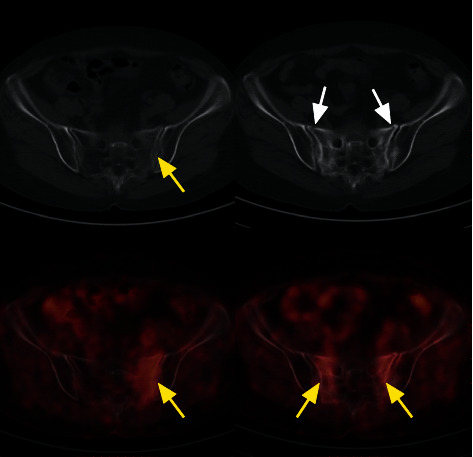
A 71-year-old woman who underwent pelvic radiotherapy for cervical cancer developed hip pain at 9 months after radiotherapy. Diffuse, mild, linear FDG uptake (SUVmax, 3.2) parallel to the sacroiliac joint in the left sacral ala (yellow arrow) was found in the fused axial image, while the low-dose CT image was normal. Follow-up FDG PET/CT at 12 months after diagnosis of SIF. The SIF became bilateral and showed FDG uptake (left SUVmax, 2.6; right SUVmax, 2.4); the low-dose CT image showed increased bone density and a new anterior sacral fracture line (white arrow).

**Figure 2:**
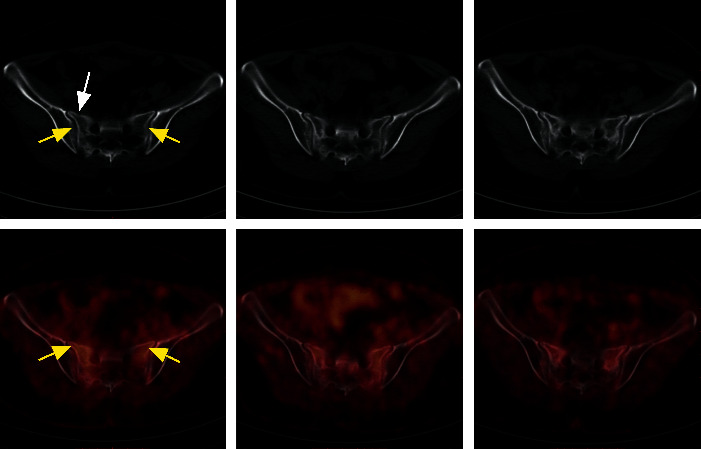
A 55-year-old woman who underwent pelvic radiotherapy for cervical cancer developed hip pain at 12 months after radiotherapy. Diffuse, mild, linear FDG uptake (left SUVmax, 3.2; right SUVmax, 3.0) parallel to the sacroiliac joint in the bilateral sacral ala (yellow arrow) was found in the fused axial image, while the low-dose CT image showed high density and a very small right anterior sacral fracture line (white arrow). Follow-up FDG PET/CT at 3 months after diagnosis of SIF. The fused image showed a slight increase in metabolism (left SUVmax, 3.3; right SUVmax, 3.5), and the low-dose CT image showed an increased bone density and high-density range. After 9 months of follow-up, the range of high density and hypermetabolism decreased; the metabolic intensity decreased (left SUVmax, 2.3; right SUVmax, 2.2), but the bone density increased.

**Figure 3:**
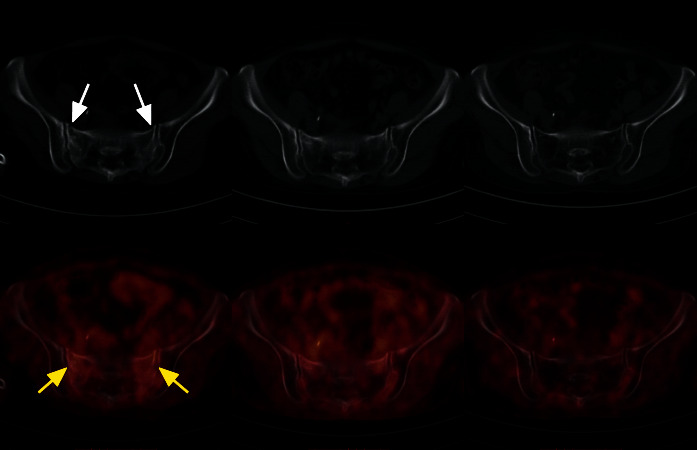
A 47-year-old woman who underwent pelvic radiotherapy for cervical cancer developed hip pain at 21 months after radiotherapy. Diffuse, mild, linear FDG uptake (left SUVmax, 3.0; right SUVmax, 2.6) parallel to the sacroiliac joint in the bilateral sacral ala (yellow arrow) was found in the fused axial image, while the low-dose CT image showed high density and a very small right anterior sacral fracture line (white arrow). Follow-up FDG PET/CT at 12 and 24 months after diagnosis of SIF. The bilateral sacral ala essentially returned to normal.

**Figure 4:**
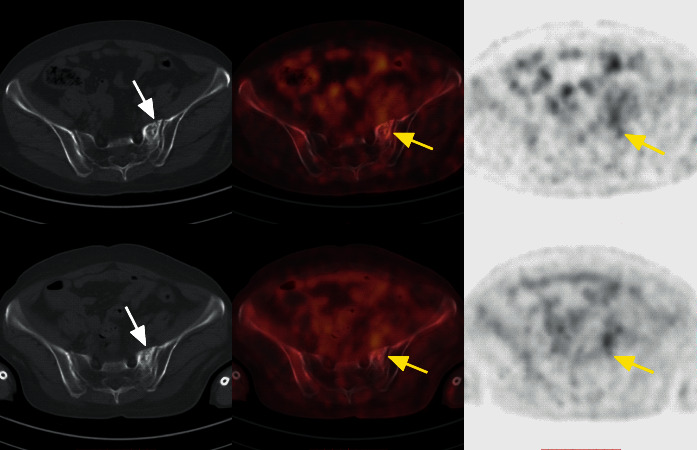
A 56-year-old woman who underwent pelvic radiotherapy for cervical cancer developed hip pain at 9 months after radiotherapy. Focal FDG uptake (SUVmax, 2.6) in the left sacral ala (yellow arrow) was found in the axial PET image, while the low-dose CT showed high density and a very small right anterior sacral fracture line (white arrow). Follow-up FDG PET/CT at 6 months after diagnosis of SIF. The bone density increased, and the metabolic intensity decreased.

In this study, 38 patients with SIF were screened; 25 had unilateral SIF (65.8%) (Figure 1), and 13 had bilateral SIF (34.2%) (Figures 2 and 3). SIF was initially found 3–42 months (median 13 months) after radiotherapy. Among the 38 patients, 35 (92.1%) showed a nonuniform increase in bone density near the sacroiliac joint with an unclear boundary around the area of increase, and the other 3 patients showed normal bone density (Figure 1); 28 (73.7%) patients showed cortical bone folds or microfracture lines perpendicular to the sacrum, all of which were located at the front of the sacrum. In all cases, metabolism was increased. The range of SUVmax was 1.8–5.9, and the average SUVmax was 3.1. Thirty-seven (97.3%) patients showed a diffuse or linear uptake pattern parallel to the sacroiliac joint, which did not completely match the area of increased bone density; the other patient showed a focal metabolic increase (Figure 4) (Tables 1 and 2).

The 38 patients were followed up for 3–59 months (mean 14 months). No patients showed malignant lesion characteristics during follow-up. Among the 25 cases of unilateral SIF, 16 cases became bilateral (64%) (Figure 1). Thirty-two of the 38 patients (84.2%) showed an increase in the extent of the lesion (density and/or metabolic abnormalities), and the lesion spread along the sacral wing margin to the center of the sacrum; 4 patients (10.5%) showed little change; 2 patients (5.3%) showed a decrease in the extent of the lesion (Figure 3). Among the 32 patients with follow-up 18F-FDG PET/CT data (6 patients with complications were excluded), 3 (9.3%) showed increased metabolism, 2 (6.3%) showed little change, and 27 (84.4%) showed reduced metabolism. Although new hypermetabolism foci occurred in some cases, the overall metabolic range decreased. During the follow-up of these 38 patients, 3 patients developed secondary infection of the sacrum or sacroiliac joint (3/38, 7.9%) (2 cases confirmed by surgery, 1 case healed after anti-infective treatment), and 3 patients developed secondary pelvic fractures and/or deformations (Table 3).

## 4. Discussion

SIF is an important cause of hip pain in the elderly [15]. Osteoporosis will increase the mechanical sensitivity and remolding of bone, leading to bone absorption exceeding bone formation and causing damage to bone elasticity and mineral content; additionally, menopausal women are at a greatly increased risk of secondary osteoporosis [16]. At the same time, elderly individuals often have muscle atrophy, which results in the loss of the protective effect of muscles on bone [17]. Radiotherapy is a common treatment for cervical cancer, but radiation can reduce the number of osteoblasts, damage the bone matrix, increase bone marrow steatosis, reduce the supply of blood vessels to bone, damage the bone microenvironment, and inhibit the repair ability of bone [6, 9]. All of the above causes lead to the main population of SIF patients being menopausal women previously treated with radiation therapy, and similar results were obtained in this study. However, young women are also at risk for secondary SIF after radiotherapy. The youngest female patient in this study was only 28 years old. By reviewing the clinical data of the patients, we found that most of the patients with SIF in this study had varying degrees of discomfort in the waist and/or hip and/or lower limbs. Therefore, we should pay attention to the occurrence of SIF in patients with lumbar and back discomfort after pelvic radiotherapy, especially in postmenopausal women [15].

Since Laurie first described sacral stress fractures in 1982 [3], epidemiological data on SIF have remained limited. Previous studies have shown that the incidence of SIF varies greatly, from 1.7% in the retrospective study by Huh et al. [18] to 89% in the prospective study by Blomlie et al. [19]. The causes of these differences may be due to different experimental designs, follow-up times, diagnostic criteria, or research subjects, but they also reflect the lack of consensus on the understanding of SIF. We did not research the incidence of SIF in this study. Firstly, SIF was affected by many factors (radiation dose, radiotherapy site, other antitumor methods, underlying diseases and patients' own conditions, etc.). Secondly, the patients included in the study required multiple follow-up visits, and many patients were excluded because of incomplete data. Finally, the onset time of some SIF may exceed our follow-up time, resulting in missed diagnosis. If the existing data are used for statistical analysis of the incidence of SIF, it will cause obvious bias. SIF can occur as early as 2 months after radiotherapy, and the course of the disease can reach 8 years or more, with a median time of 6–12 months [9]. In this study, SIF was initially found 3–42 months after radiotherapy, with a median time of 13 months and a maximum duration of 39 months. Therefore, the course of SIF is long. Some studies have shown that the incidence of radiation-induced osteitis is 83% and that the duration can reach 30 months [10]. Radiation osteitis can cause local bone damage and develop into SIF under the effect of stress, which may explain the high incidence and long course of SIF. Therefore, the occurrence and development of SIF are related to many factors [8, 9]. The accurate determination of the incidence and time of SIF requires a larger sample size and a more complete experimental design for subsequent research.

In this study, we found that, on initial diagnosis, most cases of SIF were unilateral (25/38, 65.8%) and tended to be on the left side. In most cases, the initial site of the disease was located near the sacroiliac joint of the sacroiliac wing, which should be related to the conduction of stress by the sacrum [20]. The main changes in the bone of patients with SIF were osteosclerosis near the sacroiliac joint and a blurry boundary (35/38, 92.1%); at the same time, cortical folds or fracture lines perpendicular to the sacrum were observed (28/38, 73.7%). There were 3 patients with no significant changes in the CT images of bone, but PET showed high metabolism, and in these patients, CT changes gradually appeared on subsequent follow-up. We speculate that SIF may occur in the early stage of the inflammatory response and only manifest as metabolic abnormalities, while the subsequent repair response causes an increase in bone density at the fracture site, which can then be detected by CT. Bone repair in the fracture area will cause osteoblasts and inflammatory cells to use glucose, resulting in increased 18F-FDG uptake at the corresponding site. PET can detect this change more sensitively than CT before bone density changes. It is worth noting that some of the CT manifestations of osteosclerosis and fracture lines are very slight, and it is very easy to miss these diagnoses without FDG PET and thin-slice CT of the sacrum. Therefore, when the metabolism of the sacrum is increased, we should be aware of the possibility of SIF and look for the corresponding signs (such as changes in bone density and discontinuity of the bone cortex at the front of the sacrum) on thin-slice CT. Beyond sacral insufficiency fractures, another possible sequela of radiation therapy in cervical cancer patients could be the radionecrosis of the pubic symphysis and this can also be detected by FDG PET/CT [21].

We found that when sacral metabolism returned to normal, the range of the lesion no longer expanded during follow-up, and the SIF entered a stable phase. In addition to SIF in the stabilization phase, the sacrum will show different degrees of FDG uptake. In this study, the range of SUVmax was 1.8 to 5.9, with an average of 3.1. Although metastasis also results in increased metabolism, there are some differences between them. SIF mainly manifests as diffuse or linear hypermetabolism parallel to the sacroiliac joint, with a relatively low metabolic level compared to metastasis. In this study, the majority of patients (37/38, 97.3%) showed a diffuse or linear hypermetabolism pattern parallel to the sacroiliac joint, which has also been found in other studies [11–14]. In our study, there was only one patient with small hypermetabolic foci. It may be that the SIF was in the stage of repair and stability when PET/CT was used for diagnosis, and only small areas of the lesion showed active metabolism.

On 18F-FDG PET/CT, SIF lesions tended to have a lower SUVmax than malignant lesions. Park et al. [12] showed that an SUVmax = 4.7 performed very well as the threshold value for distinguishing between benign and malignant bone lesions, with an accuracy of 88.2%. In our study, only one patient had an SUVmax higher than 4.7 (5.9). Oh et al. [13] reported the performance of 18F-FDG PET/CT in 10 cases of SIF, with an SUVmax of 2.4–7.2, including two cases showing intense uptake. The intensity of this uptake is similar to that in our cases of secondary infection. Our study shows that patients with secondary infection not only exhibited clinical symptoms and test indicators of rapid progression but also exhibited lesions often involving the sacroiliac joint and surrounding soft tissue. Although the shape and level of hypermetabolism can help us distinguish between benign and malignant lesions of the sacrum, they are not perfect standards. In clinical practice, combining the performance of FDG PET with the performance of CT facilitates the diagnosis of SIF.

In this study, the follow-up time was 3–59 months, with a median time of 14 months. Because of the lack of awareness among oncologists of SIF, the majority of patients did not receive appropriate treatment or were only given supplemental calcium and instructed to reduce their activity. Therefore, the patients in this study were in a state of natural SIF development. The follow-up data showed that the high-density range was enlarged (32/38, 84.2%) and the bone density was increased (28/38, 73.7%); only 2 patients showed a return to normal bone density after long-term follow-up. This result indicates that although SIF can be cured, cure will only be achieved in a small number of people; most patients with SIF present with persistent osteosclerosis. This result may also be because our follow-up time was insufficiently long. More accurate results obtained over a longer follow-up time are needed to confirm these findings.

Some scholars have reported that FDG uptake decreases rapidly with benign fractures and returns to normal within 2-3 months [22, 23]. In our study, we found that the pattern of metabolism was more complex than that of simple fractures. Although there was a trend of decreased metabolism and range (27/32, 84.4%) overall, the duration of FDG uptake was very long. One patient in this study was followed up after 39 months and still showed high metabolism in the sacral wing area. Compared with the baseline 18F-FDG PET/CT, we found that although the overall range of hypermetabolism was reduced, new hypermetabolism foci developed in some cases; similar results have also been found in other studies [12]. This suggests that SIF is a process of continuous bone damage and repair. During the follow-up, 64% (16/25) of unilateral SIF patients developed bilateral lesions; 6 patients (15.8%) developed serious complications (such as pelvic infection and sacroiliac joint destruction). However, the incidence of these serious complications was underestimated because some patients with SIF did not have a sufficiently long follow-up duration to determine whether adverse complications occurred. Therefore, SIF is a self-limited disease but can last for a long time, and a few patients may experience serious complications, which need early clinical intervention.

18F-FDG PET/CT can sensitively detect SIF and has value in differential diagnosis. The most important thing is to guide clinical intervention in the development of SIF. To the best of our knowledge, this is the largest study describing the 18F-FDG PET/CT manifestations of SIF after radiotherapy. This is also the first study to systematically describe the anatomical and functional changes of SIF over time. This information helps to understand SIF in depth and provides a basis for the standardized management of SIF. However, there are some limitations to the study. First, this was a retrospective study, and follow-up examinations were carried out according to the needs of tumor diagnosis and treatment, resulting in inconsistent timing of the follow-up examinations. Therefore, we could not determine the accurate onset time of SIF after radiotherapy, nor could we perform accurate dynamic follow-up examinations according to uniform time points. Second, the diagnosis of SIF was based on follow-up imaging data. Although the duration and frequency of the follow-up examinations in this study were sufficient to observe changes in other malignant lesions of the sacrum, misdiagnosis was not completely eliminated. Other limitations include the small number of cases and the insufficiently long follow-up time, which may cause selection bias.

## 5. Conclusion

It is not uncommon for SIF to occur after radiotherapy for cervical cancer, and SIF can easily be misdiagnosed as bone metastasis. Incorrect or untimely treatment can lead to serious consequences. 18F-FDG PET/CT can reflect changes in bone anatomy and metabolism, and it is an effective tool for diagnosing SIF. A small fracture line in the anterior sacrum and diffuse or linear areas of high density or metabolism parallel to the sacroiliac joint are the characteristic features of SIF. SIF is a self-limited disease that dynamically manifests as an increased bone density and high-density range and a reduced metabolic level and range. However, SIF lasts for a long time, and a few patients may have serious complications, which need early clinical intervention.

## Figures and Tables

**Table 1:** Characteristics of the 38 patients.

*Age (years)*	
Range	28–81
Average age	59.2 ± 10.6
Median age	56

*Interval from RT to SIF (months)*	
Range	3–42
Median time	13

*Follow-up time (months)*	
Range	3–59
Median time	14

*Follow-up number*	
1	3
2	7
3	16
≥4	12

RT, radiotherapy; SIF, sacral insufficiency fracture.

**Table 2:** Imaging features of the 38 patients with SIF.

*Sacral involvement*	
Left	17 (44.7%)
Right	8 (21.%)
Double	13 (34.2%)

*CT density*	
Increased	35 (92.1%)
Unchanged	3 (7.9%)
Decreased	0 (0%)

*Sacral manifestation*	
Abnormal (cortical folds or fracture lines)	28 (73.7%)
Normal	10 (26.3)

*Metabolism (SUVmax)*	
Range	1.8–5.9
Average	3.1

*Metabolic pattern*	
Diffuse or linear	37 (97.3%)
Focal	1 (2.7%)

SIF, sacral insufficiency fracture; SUVmax, maximum standardized uptake value.

**Table 3:** Follow-up imaging changes of the 38 patients with SIF.

*CT density changes*	
Increased	28 (73.7%)
Unchanged	8 (21.0%)
Increased	2 (5.3%)

*CT high-density range change*	
Increased	32 (84.2%)
Unchanged	4 (10.5%)
Increased	2 (5.3%)

*PET metabolic changes*	
Increased	3 (9.3%)
Unchanged	2 (6.3%)
Decreased	27 (84.4%)

*Complications*	
Infected	3 (7.9%)
Pelvic deformation	3 (7.9%)
None	32 (84.2%)

SIF, sacral insufficiency fracture.

## Data Availability

The datasets generated and/or analyzed in the current study are available from the corresponding author upon reasonable request.

## Abbreviations

IF: Insufficiency fracture
SIF: Sacral insufficiency fracture
18F-FDG: 2-[Fluorine-18]-fluoro-2-deoxy-D-glucose
PET: Positron emission tomography
CT: Computed tomography
SUV: Standardized uptake value
SUVmax: Maximum SUV
T/B: Target-to-background ratio
RT: Radiotherapy.
